# Delayed Calcium Normalization after Successful Parathyroidectomy in Primary Hyperparathyroidism

**DOI:** 10.1155/2021/5556977

**Published:** 2021-04-23

**Authors:** Iván Emilio de la Cruz Rodríguez, Elsy Sarahí García Montesinos, María Fernanda Rodríguez-Delgado, Guadalupe Vargas Ortega, Lourdes Balcázar Hernández, Victoria Mendoza Zubieta, Victor Hernández Avendaño, Baldomero González Virla

**Affiliations:** ^1^Endocrinology Department. Hospital de Especialidades, Centro Médico Nacional Siglo XXI, Mexico City 067200, Mexico; ^2^Faculty of Medicine, Universidad Nacional Autónoma de México, Coordinacion de Servicios a la Comunidad, Programa Comunidad Saludable-Comunidad Solidaria, Mexico City, Mexico

## Abstract

**Introduction:**

Parathyroidectomy is the curative treatment option in primary hyperparathyroidism (PHPT). The decrease of parathormone (PTH) by 50% or more from levels prior to surgery after excision predicts successful parathyroidectomy. Serum calcium is expected to return to normal within 24–72 hours after the surgery; however, nearly 10% have transient, persistent postoperative hypercalcemia. We present a case report of delayed calcium normalization after successful parathyroidectomy in a 38-year-old patient with PHPT.

**Methods:**

Parathyroidectomy was performed, with evidence of a decrease in PTH levels of more than 50% in the first 24 hours postoperatively compared to presurgical PTH; however, despite curative parathyroidectomy, a delayed calcium normalization was evidenced, with hypercalcemia persistence up to 120 hours postoperatively.

**Results:**

After the first month postoperatively, serum calcium remained normal. In conclusion, approximately 10% of patients with curative parathyroidectomy have transient, persistent postoperative hypercalcemia, which is more likely to occur in patients with higher preoperative serum calcium and PTH levels.

**Conclusion:**

Persistent hypercalcemia after the first month postoperatively is related with persistent PHPT, highlighting the importance of calcium monitoring after parathyroidectomy to predict short-term, medium-term, and long-term outcomes and prognosis.

## 1. Introduction

Primary hyperparathyroidism (PHPT) is the main cause of hypercalcemia in the outpatient setting. PHPT is a common disorder caused by the autonomous overproduction of the parathyroid hormone (PTH) attributable to hyperfunction of 1 or more glands, related with 4 different pathologic conditions: adenoma (88–90%), hyperplasia (5–7%), multiple adenoma (4–14%), and carcinoma (<1%). Most cases of PHPT are sporadic (90%). PHPT is characterized by the persistent elevation of serum calcium levels with elevated or inappropriately normal PTH levels [1, 2]. PHPT predominates among women, usually in the postmenopause, with a female/male ratio of 3 to 4 : 1. The prevalence varies by country and race [3], with a higher incidence among blacks, followed by whites, Asians, and Hispanics [4].

The clinical presentation is heterogeneous, with a spectrum from an asymptomatic disease to a hypercalcemic crisis. The diagnosis of PHPT is biochemical, and parathyroidectomy is the curative approach [1, 5].

The decrease of PTH by 50% or more from levels prior to surgery after excision predicts a successful parathyroidectomy. Serum calcium is expected to return to normal within 24–72 hours after surgery; however, approximately 10% of patients have transient, persistent postoperative hypercalcemia [6]. We present a case report of delayed calcium normalization after successful parathyroidectomy in a 38-year-old patient with PHPT.

## 2. Case Presentation

A 38-year-old man with a personal history of hypertension, primary hypothyroidism, and hydronephrosis secondary to urolithiasis was evaluated for hypercalcemia in an endocrinology department of a tertiary health center. There were no familial antecedents of hypercalcemia, lithiasis, or hyperparathyroidism. The patient reported weakness, lack of energy and strength, and mental and physical fatigue. The patient was given levothyroxine 100 mcg and candesartan 16 mg daily.

In the biochemical evaluation, laboratory features included creatinine of 1.4 mg/dL, serum calcium of 13.5 mg/dL, albumin of 4.7 g/dL, albumin-corrected calcium of 12.94 mg/dL, phosphorus of 1.9 mg/dL, vitamin D of 18 ng/mL, and PTH of 383.7 pg/mL, with evidence of hypercalcemia, hypophosphatemia, deficiency of vitamin D, and hyperparathyroidism; PHPT was diagnosed. Tc-99m sestamibi scintigraphy identified an abnormal uptake in the left inferior thyroid pole, corresponding to parathyroid adenoma.

## 3. Treatment

**Figure 1:**
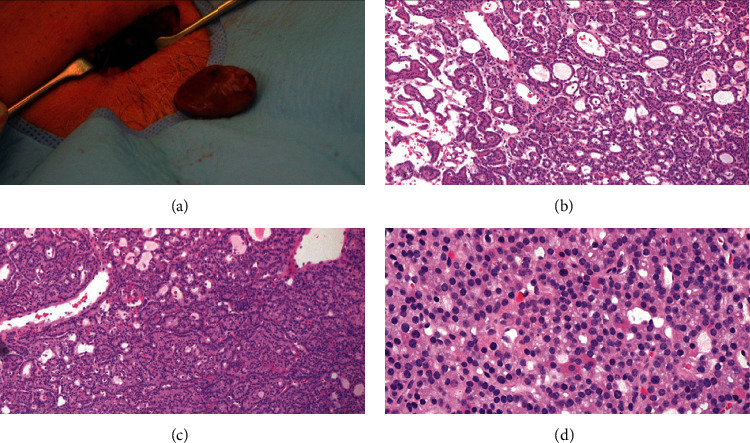
(a) Parathyroid adenoma. Hematoxylin-eosin stain (4x) showed the absence of adipose tissue and follicular (b) and trabecular (c) growth pattern, with a predominance of chief cells and some oxyphilic cells (d).

Neck exploration and parathyroidectomy were performed, with a complete excision of a tumor of 27 × 17 × 8 mm, weight of 2.3 g, and capsule integrity. An intraoperative PTH drop >50% was corroborated after parathyroidectomy, meeting the “Miami criterion.” [7] The histopathological reporting evidenced a well-circumscribed lesion with a thin fibrous capsule contained in a vascular capillary network; most of neoplastic cells resembled the normal parathyroid cells; there were uniform polygonal cells with small central nuclei and focal cystic changes, with neither atypia nor increased mitotic figures. The final diagnosis was parathyroid adenoma (Figure 1).

## 4. Outcome and Follow-Up

**Figure 2:**
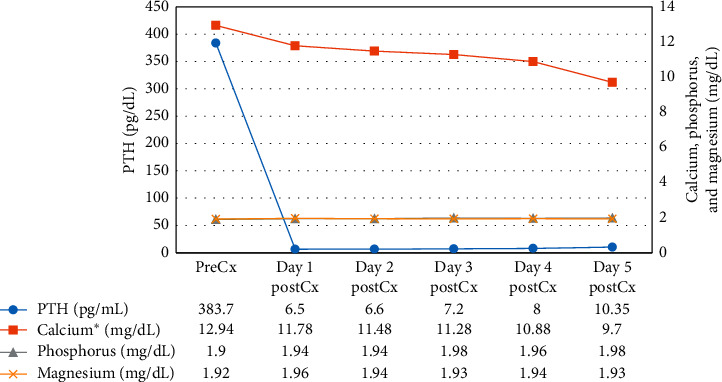
Evolution of serum PTH, calcium, phosphorus, and magnesium levels before and after parathyroidectomy. PreCx: presurgery. PostCx: postsurgery. ^*∗*^Albumin-corrected calcium.

During the first 12 hours after parathyroidectomy, a 98% reduction of PTH levels (PTH preoperatory: 383.7 pg/mL vs. PTH postoperatory: 6.5 pg/mL) was evidenced; however, a persistence of elevated serum calcium (12.1 mg/dl) was observed. Magnesium remained normal during evolution. During the first 48 hours after parathyroidectomy, the patient received 1500 ml of 0.9% sodium chloride solution I.V. and 500 ml of water P.O. After this time, he continued with 2000 ml of water P.O. during his follow-up. In the clinical follow-up, the evidence of hypercalcemia and normal PTH levels persisted up to 96 hours, with a delayed calcium normalization up to 120 hours (Figure 2). After the first month postoperatively, serum calcium remained normal; however, the patient currently continues with a strict monitoring of serum calcium and PTH levels.

## 5. Discussion

We presented a case report of a patient with symptomatic HPTH, who underwent surgery for the presence of renal involvement, age younger than 50 years, and serum calcium level greater than 1 mg/dL above normal, with the histopathological evidence of parathyroid adenoma. We highlighted that, despite a successful parathyroidectomy, serum calcium levels remained elevated for a longer time than reported in the literature, with a delayed calcium normalization up to 120 hours.

Parathyroidectomy is the curative and definitive treatment of PHPT, and it is indicated for all patients with symptomatic PHPT, evidence of renal involvement, osteoporosis, fragility fracture, vertebral fracture, when the serum calcium level is greater than 1 mg/dL, age of 50 years or younger, and when parathyroid cancer is suspected [1]. Parathyroidectomy should be conducted by surgeons with adequate training and experience.

The “Miami criterion” is defined as a PTH decrease >50% from either the highest preincision or pre-excision hormone level in a peripheral blood sample obtained 10 minutes after complete excision of all hyperfunctioning parathyroid tissues. After this, an intraoperative PTH decrease >50% occurs, and the observed hormone dynamic guides the termination of the operation without further exploration. If the 10-minute sample does not meet the criterion, a delayed sample at 20 minutes is measured, and/or further neck exploration is continued until all hypersecreting parathyroid glands are removed, confirmed by another >50% decrease from the highest subsequent pre-excision sample. This protocol was developed and refined at the University of Miami and indicates a successful parathyroidectomy, with a sensitivity of 98%, specificity of 97%, positive predictive value of 99%, negative predictive value of 90%, and overall accuracy of 97% [7].

In the hands of an experienced surgeon, cure rates for parathyroidectomy in sporadic PHPT approach 95% to 99%, with a low complication rate (<1–3%) [1, 5]. Curative parathyroidectomy is defined as the normalization of calcium homeostasis lasting a minimum of 6 months after surgery [1].

Currently, there is no consensus about the time that serum calcium normalizes after parathyroidectomy. After a successful parathyroidectomy, serum calcium levels normalize and reach a nadir at 48–72 hours postoperatively [8, 9]; however, approximately 10% of patients have transient, persistent postoperative hypercalcemia, which is more likely to occur in patients with higher preoperative serum calcium and PTH levels. Most patients with PHPT have normal serum calcium levels within the first two weeks after parathyroidectomy, and 96% will have normal serum calcium levels in the first postoperative month. Hypercalcemia that does not normalize within the first 30 days postoperatively has been associated with persistent PHPT [6], which is defined as a failure to achieve normocalcemia within 6 months after parathyroidectomy [1].

The early biochemical response of PTH and serum calcium levels after parathyroidectomy is the main predictor of persistent or recurrent PTHT after 6 postoperative months. Patients with a partial biochemical response after parathyroidectomy have a greater risk of recurrent hypercalcemia compared to those with complete biochemical response. The postoperative presence of persistently increased PTH but normocalcemia or persistently increased calcium but normal PTH increases 1.6 and 2 times, respectively, the risk of recurrent hypercalcemia [10]. Despite these data, there has been no demonstration of the association between transient hypercalcemia after parathyroidectomy and persistent or recurrent PHPT [6]. The role of transient hypercalcemia and the delayed calcium normalization in the evolution and prognosis of the patient with PHPT represents an important and relevant research topic for long-term studies.

## 6. Conclusion

Approximately 10% of patients with curative parathyroidectomy have transient, persistent postoperative hypercalcemia, which is more common in patients with higher preoperative serum calcium and PTH levels. Most of these patients achieve calcium normalization within the first postoperative month. Hypercalcemia after the first 30 days postoperatively is related with persistent PHPT, highlighting the importance of calcium monitoring after parathyroidectomy to predict short-term, medium-term, and long-term outcomes and prognosis. There is a lack of information about the association between transient, persistent postoperative hypercalcemia and the development of persistent PHPT; future long-term studies about this research topic are necessary.

## Data Availability

All the data used in this case are available from the corresponding author upon reasonable request.
